# “The future”: Interpretative phenomenological analysis of general practitioners' experiences of co‐employed clinical psychologists

**DOI:** 10.1002/jgf2.774

**Published:** 2025-02-24

**Authors:** Katie Monnickendam, Peter Keohane, Rebecca Magill

**Affiliations:** ^1^ Aneurin Bevan University Health Board Caerphilly UK; ^2^ University of Plymouth Plymouth UK; ^3^ Faculty of Health University of Plymouth Plymouth UK; ^4^ Launceston Medical Centre Launceston UK

**Keywords:** clinical psychologist, general practice, interpretative phenomenological analysis, primary healthcare

## Abstract

**Background:**

General practice is overwhelmed and understaffed. New models and modalities must be considered from the front door of healthcare. Like general practitioners (GPs), clinical psychologists are qualified to work across the age range and transdiagnostically.

**Aim:**

To explore GPs' understanding of the role of a clinical psychologist and to examine what is helpful and unhelpful for GPs about working alongside a clinical psychologist within general practice.

**Design and Setting:**

This research took place within a primary care general practice in the United Kingdom.

**Method:**

Seven qualified GPs were recruited as participants using a purposive sampling method. Interpretative phenomenological analysis was used to analyze participant's experiences of working alongside clinical psychologists.

**Results:**

Three superordinate themes and 12 subordinate themes were identified. First, GP clinical psychologists support patients directly by providing preventative care, reducing stigma, and offering a different perspective. Second, they support GP well‐being and contribute to emotional and behavioral changes in staff. Third, they help to alleviate pressures on wider systems, ease navigation of external services, challenge dominant systems within healthcare, and support community interventions.

**Conclusion:**

GP clinical psychologists impact general practice in multiple ways: indirectly through patient care, by GPs themselves, and by addressing wider systems. Future research is encouraged to explore the perspectives of other staff members and patient's experiences.

## 1 INTRODUCTION

General practitioners (GPs) are the first contact for many patients with mental health difficulties.^1^ In the United Kingdom, this number is estimated to be one in four.^2^ GPs have limited time to assess patients, decide on interventions, and make referrals.^3^ This causes significant pressure and many appointments overrun.^3^ The direct consequence is GPs working through their breaks, staying late, and impacting their well‐being.^4^

In addition, GPs have reported that while they feel skilled in managing common mental health difficulties, this is not true for more complex presentations.^5^ Mental health placements are not compulsory in the United Kingdom^6^ and it appears that the GPs who have received mental health training have done so in addition to their core training. This accounts for less than half of GPs.^7^ Given their increasing prevalence, especially following the COVID‐19 pandemic,^8^ mental health presentations will progressively become a significant part of general practice, placing additional pressure on a system already at capacity. Workload has already been identified as the biggest concern,^9^ and to cope with the impact of this, many GPs choose to reduce their working hours, take a career break, or opt for early retirement.^10^

Practices across the United Kingdom are increasingly faced with recruitment and retention difficulties, with one in seven posts vacant.^11^ The British Medical Association reports there are 1877 fewer qualified full‐time GPs working in England than in 2015.^12^ The number of patients per GP has increased by 7.1% since 2019, currently sitting at an average of 2292.^13^

In the United Kingdom in the 1990s, it was more common for clinical psychologists (CPs) to provide therapies within general practice settings.^14^ Improving Access to Psychological Therapies (IAPT^15^) and austerity^16^ then restructured both mental health services and primary care, resulting in the exit of CPs from general practice. CPs are unique in their offer of both direct and indirect interventions and undergo doctoral‐level training enabling them to work across the lifespan with a range of presentations.^14^ Direct interventions involve psychotherapeutic interventions with clients. Indirect interventions include reflective practice, staff training, consultation, supervision, and system‐based intervention and service development.

GP practices do not have inclusion and exclusion criteria unlike many mental health services.^1^ It is likely that CP intervention from general practice would allow for a preventative model of mental health, with difficulties being identified and managed earlier on, and help joining up services, particularly between primary and secondary care, and physical and mental health.

Given the possibilities, it is surprising that there are only a handful of relevant studies on CPs working in a GP context. The literature is heavily focused on direct interventions, mainly therapy provision, often not considering indirect interventions. Studies that did acknowledge these suggested that consultations to upskill staff,^17^ providing feedback on onward referrals,^18^ and providing resources for staff while improving their awareness of signposting services^19^ were helpful.

Vines et al.^20^ reported patient's mental health symptoms reduced following direct intervention with CPs in general practice. Abrahams and Udwin^19^ reported a reduction in waiting times when compared to secondary care services, allowing for earlier intervention. Another study highlighted the importance of CPs being embedded within the team,^18^ allowing for the sharing of psychological ideas and informal consultation, as well as feedback on referrals. Dath et al.^17^ described the relationship as bi‐directional, emphasizing a continuous learning approach. Finally, several studies referred to the reduced pressure on other services^17^, ^19^, ^21^ and the reduction in stigma.^18^, ^19^ While this evidence hints at the effectiveness of CPs within general practice, it also highlights a literature gap regarding a mixed intervention model.

The Royal College of General Practitioners (RCGP) policy paper states that health outcomes are improved by multidisciplinary working that is “local, accessible and familiar,”^22^ ^, p. 6^ This way of working is likely to build trust and promote effective relationships. Basing CPs in general practice could support this.

Several case studies have recently reported on the effectiveness of embedding CPs into primary care.^23^ Although the projects operated in different ways, the evaluations highlighted the benefits of GP CPs. These included providing a service for patients deemed “too complex” for IAPT, but who do not meet referral criteria for secondary care, access to specialist advice for primary care staff supporting patients with complex difficulties, and early intervention as a preventative approach for the deterioration of mental health difficulties.^14^ In addition, the economic implications of these services were very promising.^14^

A recent article interviewed a GP CP^24^; however, none explored in‐depth GP experiences of working alongside a CP. This gap highlights an opportunity to qualitatively review and provide rich experimental data on the impact of CPs working in general practice. A GP practice in the United Kingdom employed a CP full‐time in February 2021. The role of the CP within this practice is to provide applied psychological interventions, both directly and indirectly with staff, offering consultations, training, and reflective practice. The role also involves leadership of community and systemic interventions. The research, therefore, sought to address how GPs experience the role of the CP within their practice and what were the helpful/unhelpful aspects of working together.

## 2 METHOD

### 2.1 Design

Interpretative Phenomenological Analysis (IPA^25^) was chosen to capture the rich, experiential data of GPs' experiences of working alongside a full‐time CP. IPA is helpful when little or no research has been completed^26^ and offers rich, enhanced quality data.^27^

### 2.2 Participants

With the chosen analysis method, a small number of participants is recommended, and a number between six and eight is generally considered appropriate.^25^ The researchers, therefore, aimed to recruit seven participants. This accounted for half of the GPs in the practice at the time. All participants were qualified GPs who were employed by the practice in the United Kingdom and had been working alongside the CP since February 2021. A purposive sampling method was used for recruitment, and pseudonyms were allocated.

### 2.3 Interview structure

Semi‐structured interviews were designed in consultation with two CPs who were not involved in the research. The interview schedule consisted of seven questions (Appendix S1), with an option at the end to bring anything else the participant felt was appropriate.

### 2.4 Procedure

Recruitment took place between 2022 and 2023. All qualified GPs were approached via an email from the practice manager detailing the research. Seven participants responded and were interviewed in a private room at the practice. Participants were reminded that they could stop the interview at any time, and after the interview, were provided with a debrief sheet. The interviews were audio‐recorded.

All interviews were transcribed and analyzed using the seven‐step framework of IPA outlined by Smith et al.^25^ Each transcript was analyzed in turn, and exploratory notes were recorded, allowing for the construction of experiential statements. Subsequently, connections across the experiential statements were identified, and personal experiential themes were named and organized. This process was repeated for all transcripts and then group experiential themes were identified. These were shared with participants to check for resonance with their experiences.^28^

To account for researcher biases, bracketing interviews with peer trainees were conducted during data collection, transcription, and analysis stages, allowing participants' experiences to remain at the core of the analysis process.^29^ Extracts from transcripts were shared in an IPA research group to review and discuss exploratory notes and experiential statements. The primary researcher recorded a reflexive journal throughout the process to further account for bias.

### 2.5 Ethics

This research was approved by the sponsoring university and the NHS Health Research Authority.

### 2.6 Researcher position‐written by author KM

As a clinician, I have experience working in an IAPT service for 2 years. While this service was well run and designed to provide many individuals with access to psychological support at a primary care level, I became increasingly concerned about the idea of a “one size fits all” model. While the predominant therapy modality, cognitive behavioral therapy (CBT) was well suited to many individuals, there was a substantial number for whom this was not the case. It was these individuals who would disengage, be discharged from the service, and get referred back to their GP. While I imagine this may have been frustrating for these individuals and may have generated feelings of hopelessness and isolation, I rarely considered the impact this had on GPs. Given that GPs were likely referring the patient for support they could not or did not feel able to provide, I can imagine similar feelings of frustration and hopelessness being generated in GPs when patients were referred back to them. I reflected that GP voices are often lost within patient care and hope that this research is a forum that will allow these voices to be heard.

Since starting the research process, I was offered a placement at the practice as part of my doctoral training. Although I had started the placement in the middle of the research process, this experience has helped to shape my research. It has allowed me to be a part of the “other side” of what was described above and has really emphasized the importance of hearing the voices of GPs, as well as other multidisciplinary professionals.

Given my position in the research, processes such as bracketing and keeping a reflexive journal ensured my analysis remained independent and reduced the potential for bias.

## 3 RESULTS

Three superordinate themes and 12 subordinate themes emerged. Participants recognized the impact on themselves, patients, and the wider system. These are outlined in Table 1.

**TABLE 1:** Overview of themes.

Superordinate themes	Subordinate themes
1. Impact on patient care	1.1 Early intervention is key 1.2 There is less stigma in primary care 1.3 An attachment‐informed lens 1.4 A different perspective
2. Impact on GPs	2.1 Internal process change 2.2 External process change 2.3 GP well‐being
3. Impact on wider systems	3.1 Alleviating pressures 3.2 Challenges to the medical model 3.3 Ease of navigating 3.4 Hierarchies and disruption 3.5 Community intervention

1. Impact on patient care:
  This superordinate theme represents participants' experiences of how employing a CP can impact patients directly. Early intervention approaches, having an environment that is less stigmatizing than general mental health settings, considering patient–doctor relationships, and thinking about things a bit differently were all thought to positively impact on patient care. This talks about the ease of patients accessing psychological services in the first place, as well as being able to address and support them within this setting.
  1.1 Early intervention is key:
    Most participants recognized that increasing mental health waitlists has a negative impact on patients' difficulties, and early intervention leads to good outcomes. David and Emma focused on how the current model, a reactive approach, pushes clinicians toward the medical model. Other participants compared the new approach with co‐located CPs to external services. Fred shared his experiences of an ADHD service and the consequence of not being able to seek advice from the CP: “that referral would have gone the wrong way and then it would have sat on the adult's system for 3 years.”
  1.2 There is less stigma in primary care:
    Most participants recognized that patients were more willing to access psychological support from general practice. They reflected that the environment and process were less stigmatizing. The informal nature of being referred to the in‐house CP was important. Eric focused on the lack of CP exclusion criteria in‐house, reiterating the narrative that this may reduce stigma.
  1.3 An attachment‐informed lens:
    Attachment is used to describe “psychological connectedness between human beings,”^30^ ^, p. 194^. In this case, it describes the patient–doctor relationship, which participants acknowledged often takes time to develop. Time that GPs are limited on and connection that is not valued by the medical model. David and Paul felt patients were more likely to consider mental health with someone whom they had a good relationship with. Emma reflected on difficulties that come from a poor GP–doctor relationship: “…patients getting very dependent on coming to see the GP.”
  1.4 A different perspective:
    Most participants recognized that CPs offer a different way of thinking, especially with overlaps between physical and mental health. Eric reflected on a shared understanding: “I've seen they've been sort of been fairly carefully dissected […] and the explanation for their chronic, uncurable pain becomes more apparent […] it certainly helps me, but … it will help the patient as well.” This talks about a balance of diagnosis and psychological formulation. Paul considered how working alongside CPs invited different types of information and bi‐directional learning
2. Impact on GPs:
  This superordinate theme focuses on the personal and professional impacts the CP had on GPs themselves. This encapsulates both personal effects as well as changes to their professional practice.
  2.1 Internal process change:
    Participants described cognitive and emotive shifts. The majority reflected shifts in recognizing when a patient's needs are going to be better supported by a CP. Paul recognized the limitations of GP knowledge: “it doesn't matter if you've done effectively [a] minimum of 10 years training to do general practice, that doesn't mean that you're the best person for everything.”
    Furthermore, participants reported a shift in their understanding of the CP role, as well as internal narratives about psychology. All participants shared positive narratives about the CPs and considered how this could affect practice, with staff being more likely to reach out to other mental health professionals. Participants stated psychological support should be a core part of the GP working model.
  2.2 External process change:
    Participants reflected that they felt more confident in discussing mental health difficulties with patients. David and Eric reflected on access to more resources, while others highlighted the joint working opportunities. However, Sophie considered these may be threatening for patients.
    Participants reflected on the impact of informal CP consultations on their practice. Several noticed a reduction in their mental health prescribing: “…my immediate feeling is that mine will be a fraction of where it was” (Fred).
  2.3 GP well‐being:
    Participants reflected on the impact on their well‐being. Most found that CPs sharing the workload was helpful, particularly with emotionally challenging work. Mel identified the CP as: “that person who can, I think, lift a bit of the burden from me.” There is an acknowledgment that the CP reduces workload. Sophie further described feelings of safety in the relationship, suggesting the importance of secure staff attachments within general practice, and David described the CPs as “a safe pair of hands.” Participants felt that this trust and supportive relationship encouraged them to ask questions and seek advice about patient care through consultations. Even knowing that the CP was available seemed to make participants feel more relaxed, likely indirectly affecting patient care as well as potentially improving staff retention. Additionally, participants reported that workplace well‐being improved since the CP was introduced. Here, we note that GP's well‐being is improved via a workload reduction as well as via promoting a safe, containing place to work within. Both may well have the capacity to influence recruitment and retention of GPs.
3. Impact on wider systems:
  This superordinate theme is about wider system impacts of employing a CP. Participants' experiences reflected how systems focus on the medical model and how this may change following the employment of a CP. Participants also reflected on the influence CPs can have on wider systems and their own practice.
  3.1 System pressures:
    Participants recognized the impact of system pressures and service gaps. Emma and Fred highlighted the shortage of GPs and third‐sector mental health support. They felt that system pressures affected their ability to do their job and deliver good patient care.
    Other participants noticed changes over time and considered the consequences for services. Emma stated: “the mental health team have gone from being a supportive organisation to being a crisis organization […] that's left massive gaps in what we can achieve.” Others reflected on not knowing onward referral criteria and shared frustrations as provision and remit changed so rapidly, highlighting service gaps between IAPT and secondary mental health. Some participants had concerns about other services relying on GP CPs.
  3.2 Challenges to the medical model:
    Most participants reflected on their reliance on the medical model. They attributed this to it being a core part of training, as well as having worked in this way for several years. Several participants commented on how mental health does not “fit” into the medical model: “mental health […] takes time, and it doesn't fit nicely into a 10‐minute consultation” (Mel). Participants reflected frustration around this, knowing that internally they are challenging the medical model and its application.
    While considering alternative models may be valuable, participants also identified disadvantages such as a sense of uncertainty or unfamiliarity. Also, while participants recognized the CPs as “experts” in mental health, this increases the risk of power imbalances and contributes to the idea that only specialists can do mental health work.
  3.3 Hierarchies and disruption:
    Participants reflected on the hierarchical nature of general practice and spoke of welcome disruption to this. They recognized that GPs are often part of the medical hierarchy and reflected that the introduction of CPs influenced this. This seemed to relate to the doctoral qualification being well respected. David considered CPs to be equal to GPs: “I think because you're seen as an equal, you're not delegating down, you're delegating sideways.”
    Other participants considered how CPs can influence change within management. Mel considered the CP as one of the clinicians as well as part of management, suggesting that CPs have the power to influence general practice.
  3.4 Ease of navigating the wider system:
    Participants shared frustration and confusion in the regular changes to mental health services. Fred described the elusive nature of them: “as a GP on the outside, they're so opaque, there's just no way of even learning how, unless you've been in it, you can't work out how to get anyone in.” There were also feelings of “stuckness,” with not having any other option but to refer and wait on the list. Mel reported feelings of frustration at how misunderstandings in service provision often result in rejected referrals. Participants recognized the impact this had on their practice and patient well‐being.
    However, having the CPs present radically changed this, with participants reflecting on improvements in their understanding of different services. Paul described the CP as a “pathway navigator” and valued having someone who knows the systems. Eric considered how the CP's professional relationships with other services eased this process.
  3.5 Community intervention:
    Participants reflected on how CP input could impact the local community. Some noticed how CPs introduced systemic formulations in patient care, considering the individual in the context of their experiences and relationships with others. Others highlighted how CPs make connections throughout the community. David reflected on thinking holistically about patients.
    Participants also described how the practice, and its stakeholders, had embraced the change from the exclusively GP dominant model. Mel reflected on the role of allied healthcare professionals working within the practice, which was supported by Paul: “it's foot in the door for recognition of, of a…a more, a more MDT approach….”

### Interconnectedness

Each of the themes lends evidence to the benefit of CPs in GP; however, the whole is greater than the sum of the parts. The interconnectedness of each theme to another is important. For instance, the reduction in stigma will likely improve early intervention and both themes here will have an impact on GP workload, aside from the burden being lifted by a CP completing some of the work themselves.

These relationships between themes represent systemic efficiencies where the currently overstretched GP system might change current problematic patterns into virtuous cycles generating sustainable systems.

## 4 DISCUSSION

### 4.1 Findings

This research explored GP experiences of working alongside a CP in the general practice setting. The findings suggest impacts on patient care, GPs, and wider systems. GPs described CPs working preventatively, providing different perspectives to patient difficulties, with potential to reduce stigma around accessing mental health support. GPs reflected on changes in the way they think and feel about their work, how they approach it, and improvements in their well‐being. GPs also reported the CP holding in mind the changes in external systems and forming good relationships to support navigation and community intervention. They considered how CPs can sit within the medical model as well as challenge it and they experienced the CP to be part of the team as well as a member of management, thus disrupting the hierarchical nature that is common within general practice.

This research complements previous research, acknowledging the importance of CPs offering direct intervention within this setting. This research adds to previous studies considering the indirect approaches that CPs can offer. It reported on the positive impact on staff well‐being, increased GP confidence in managing mental health difficulties, and challenges to the dominant narrative of the medical model. This may positively impact patient care, as well as potentially address the retention and recruitment difficulties identified by Osbourne (11 by considering GPs' attachment and well‐being as well as by reducing work burden). In addition, GPs reported positive narratives about psychology and psychological professionals, which could influence their working relationships with mental health professionals. Therefore, in addition to the direct interventions stated above, this research suggests the CP role should also support indirect approaches.

The RCGP policy paper emphasizes therapeutic relationships and attachment to improve patient care and GP experience. This was supported and enhanced by these findings, suggesting a GP‐based CP that is “local, accessible and familiar,”^22^ ^, p. 6^ should be part of the general practice multi‐disciplinary team.

This research reiterated ideas that GPs appreciated having a trained professional whom they could make referrals and trust to support patients. GPs also valued the offer of joint sessions with the CP and reflected on the positive impacts on patient care, as well as their own learning and understanding of mental health.

Similarly to Abrahams and Udwin,^19^ who consider the reduced waiting times with CPs based on general practice, this research highlights the early intervention approach that is enabled by GP CPs. This is likely to impact positively on patients and have wider impacts such as reducing the burden on other services. Moreover, earlier intervention may mean that fewer sessions with a CP are required.

This research supports previous ideas about this model reducing the stigma of mental health support.^18^, ^19^

Although previous studies have made brief reference to the impacts on wider systems, this research is unique in considering these further. GPs reported finding it easier to navigate external mental health services with CP support. They also reflected on their reliance on the medical model and considered broadening their own approach. In addition, this research reports on how CPs based within general practice can gently disrupt the hierarchical nature of general practice to help broaden perspectives.

Ultimately, this research supports the recent BPS report^14^ stating that CPs should be embedded into general practice. Clinically, GPs feel more confident in managing mental health difficulties and have the opportunity to work jointly with the CPs and benefit from this. Participants summarized the findings succinctly stating that GP CPs are “needed and important” (Sophie) and “it's the future” (Paul) of general practice. Their enthusiasm conveys that GP CPs are an important area to invest in, and future research and developments would be highly valued.

### 4.2 Limitations

This research recognized that there are fewer findings about the unhelpful aspects of working alongside a CP compared with the other research aims. This was something that participants found difficult to answer and often reframed into ideas about there not being enough CPs in general practice. In addition, there have been national struggles with GP recruitment. This may have influenced GPs to promote a positive, psychologically informed environment in their responses in order to aid and encourage recruitment.

This research focused singularly on GP perspectives. Within the practice, there is a multidisciplinary team including nurses, paramedics, and social prescribers. The viewpoints of these professionals, as well as practice managers, administration staff, and patients, would be an important perspective to obtain.

These data were restricted to one practice but as the GP CP role develops, further research in other practices is welcomed.

Finally, the field researcher and co‐author for this research was a CP employed by the practice. In addition, the primary researcher also had a placement at the practice during the time of the research. While efforts were employed to mitigate the effect of this, it must be considered as a limitation.

### 4.3 Implications

This research has several implications for clinical practice and research.

In terms of practice direction:

- GP CPs can benefit general practice and add to the current models of working.
- GP CP's job description should include a mixture of direct work with patients, indirect support, and systemic activities.
- Indirect support might involve supporting staff well‐being, offering reflective practice groups, and offering training.
- Systemic activities might include policy, recruitment, formulating system pressures, and bringing psychosocial perspectives to solution finding.

In terms of future research:

- Perspectives of other staff members in general practice should be explored.
- Perspectives of patients accessing psychological support within general practice should be explored.
- Future research should take into account what can be learned from the pre‐IAPT CPs who worked in general practice.
- Detail could be sought, in future research, on the challenges of working with CPs or of CP–GP integration.

## 5 CONCLUSION

This study aimed to provide insights into GPs' experiences of working alongside a CP. The findings build on previous literature by emphasizing the far‐reaching impact that this model within general practice can have. It considers impacts on patient care, GPs, and the wider systems.

## CONFLICT OF INTEREST STATEMENT

Author R.M. was employed by the general practice used for the research and author K.M. had a placement at the practice during the time of the research. The funding for this study was provided by the University of Plymouth in partial fulfillment of the degree of Doctor of Clinical Psychology (DClinPsych). The funding source had no role in the design, practice, or analysis of this study. This research was approved by the sponsoring university and the NHS Health Research Authority.

## ETHICS STATEMENT

Ethics approval statement: This research was approved by the sponsoring University and the NHS Health Research Authority.

Patient consent statement: Participants who chose to participate completed a written consent form outlining the process of participation and ability to withdraw.

Clinical trial registration: This was not a clinical trial so registration not applicable.

## Supporting information


Appendix S1.
