# Supplementary material for: “The future”: Interpretative phenomenological analysis of general practitioners' experiences of co‐employed clinical psychologists
Source: J Gen Fam Med. 2025 Feb 24;26(3):255–62. doi: 10.1002/jgf2.774 (PMC12022423; doi:10.1002/jgf2.774)
Supplement: Supplementary file 1 — Appendix S1. [file JGF2-26-255-s001.docx]

Appendix 1: Interview questions

***NOTE***: We appreciate that you may have personal experiences of seeing or working with a clinical psychologist, but for the purposes of this research we are interested in your professional experience of working alongside the GP Psychologist.

1. What do you understand the role of the GP psychologist to be?
2. How has it come about: why here, and why now?
3. GP Psychologists offer a range of support such as supervision, consultation, training, and reflective practice in addition to direct work with patients. Have you used any of these services or observed any of these services being used? Please describe your experiences of them.
4. What is most helpful about having a GP psychologist working in the practice?
5. Are you able to give examples of ways in which, as a result of having the GP Psychologist:
   1. Your own practice has changed?
   2. Things in the practice have changed?
   3. Your patient’s health and wellbeing has changed?
6. Have you any thoughts about how you think the role of the GP psychologist could be improved or developed?
7. Would you recommend a GP psychologist to other practices? How might you suggest or recommend this model is implemented?
